# Oral and Maxillo-Facial Manifestations of Systemic Diseases: An Overview

**DOI:** 10.3390/medicina57030271

**Published:** 2021-03-16

**Authors:** Saverio Capodiferro, Luisa Limongelli, Gianfranco Favia

**Affiliations:** Department of Interdisciplinary Medicine, University of Bari Aldo Moro, Piazza G. Cesare, 11, 70124 Bari, Italy; gianfranco.favia@uniba.it

**Keywords:** oral cavity, head and neck, systemic disease, oral signs of systemic diseases, early diagnosis, differential diagnosis

## Abstract

Many systemic (infective, genetic, autoimmune, neoplastic) diseases may involve the oral cavity and, more generally, the soft and hard tissues of the head and neck as primary or secondary localization. Primary onset in the oral cavity of both pediatric and adult diseases usually represents a true challenge for clinicians; their precocious detection is often difficult and requires a wide knowledge but surely results in the early diagnosis and therapy onset with an overall better prognosis and clinical outcomes. In the current paper, as for the topic of the current Special Issue, the authors present an overview on the most frequent clinical manifestations at the oral and maxillo-facial district of systemic disease.

## 1. Introduction

Oral and maxillo-facial manifestations of systemic diseases represent an extensive and fascinating study, which is mainly based on the knowledge that many signs and symptoms as numerous systemic disorders may first present as or may be identified by head and neck tissue changes. Dentists and general practitioners play a key role in such early identification; therefore, the clinical examination of the oral cavity should be performed accurately avoiding unexplored sites and a detailed medical history mandatorily collected. Doubtful lesions of the oral mucosa, lip, skin of the face and neck, neck and sub-mandibular swellings, atypical radiological images or occasionally founded, atypical coloration and forms of the teeth, unexplained and/or sudden mobility of one or more teeth, unexplained bleeding from gingiva, oral mucosa and nose must be always careful investigated and monitored to achieve a medical explanation. A cytological scrape or an incisional biopsy of suspicious lesions or a fine needle aspiration of doubtful swellings with subsequent cyto-histological examination must be performed whenever deemed necessary to obtain a definitive diagnosis.

We review the most common and changeling oral and maxillo-facial findings possibly associated to or manifested at the onset of systemic diseases in adult and pediatric patients, along with diagnostic criteria and indications about differential diagnoses. 

## 2. Infectious Diseases

**Figure 1:**
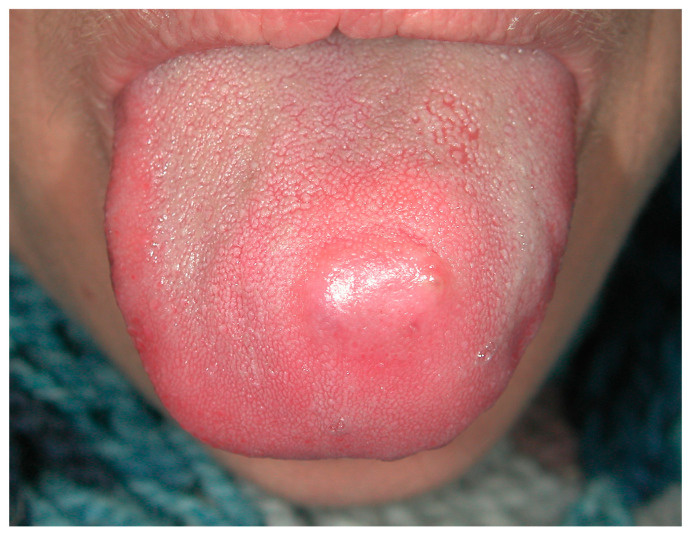
Tongue swelling with pus discharge diagnosed as actimomycosis by a fine needle/core needle biopsy.

Actinomycosis is an infrequent bacterial chronic infection and the disease most misdiagnosed by experienced clinicians too. It is sustained by *Actimomyces* (mostly *Actinomyces israelii* or *naeslundii*), a saprophytic component of the endogenous flora of the oral cavity, and is anatomically and clinically divided into three types; cervico-facial, pulmonary, and abdominal-pelvic [1,2]. The cervicofacial type is the most frequent and may cause, especially in immunocompromised individuals, suppurative and granulomatous inflammatory lesions, with a locally aggressive and destructive behavior [3,4]. Soft tissue swelling (Figure 1) or osteomyelitis with pus discharge, sinus tract formation are common clinical findings, resembling the classical symptoms of abscesses, acute sialoadenitis and lymphoadenitis. Periapical localization may look like conventional periapical lesions (granuloma, cysts) in an x-ray, but it remains essentially unresponsive to medical and instrumental treatments. Microbiological cultures and histopathology are mandatory for diagnosis of this disease which, if untreated, may develop pulmonary, intracranial and para-pharyngeal diffusion [3,4,5].

Tuberculosis is a chronic granulomatous disease, typically of the lungs, but potentially involving multiple body systems, with the higher prevalence in developing countries or among immunocompromised patients, which carries a substantial rate of mortality [6]. Extra-pulmonary involvement mainly occurs due to endogenous spread of the pathogen from the primary site. The primary form arises from direct inoculation of the infecting pathogens in the susceptible sites in absence of systemic involvement, like in soft tissues of the oral cavity and the jawbone, mostly the mandible, where possible routes of infection are both sites of recent dental extraction, mucosal wounds or lacerations, and systemic hematogenous and/or lymphatic spread [7]. Clinically, tuberculosis may resemble periodontal or periapical lesions or abscesses with or without intra- or extra-oral draining, or as an impressive ulceration of oral mucosa associated or not to cervical lymphadenopathy [6,7,8].

**Figure 2:**
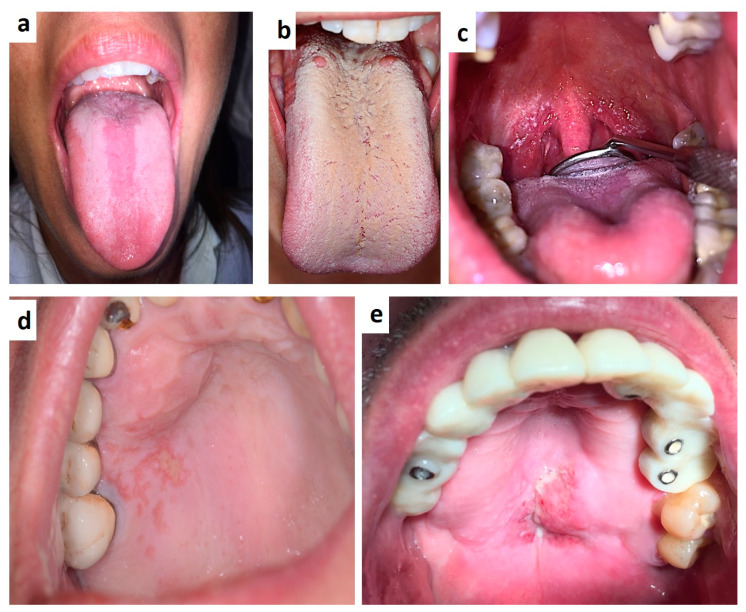
(**a**–**e**) Oral candidiasis of the median tongue (**a**), diffuse (**b**) or oro-pharingeal are frequent the first sign of HIV infection in unaware patients, as well Herpetic lesion of long-lasting duration occurring in un-conventional site, like the palate (**c**). Non-Hodgkin’s lymphoma of the palate in an already diagnosed HIV-patient.

Human immunodeficiency virus infection is nowadays the fourth leading cause of death worldwide [9] and oral manifestations are frequently the earliest signs, especially in unaware patients or detectable in 30%–80% of already diagnosed patients [9,10]. Oro-pharyngeal candidiasis and hairy leukoplakia are the most common early findings in undiagnosed cases [11,12,13]. Also, oro-maxillo-facial signs may include manifestations of bacterial, viral and fungal infections (mostly sustained by Mycobacterium, HPV, HSVs, CMV, Cryotococcus, Histoplasma, Aspergillus, Mucoraceae, etc.), (Figure 2a–d) pseudo-tumoral lesions (necrotizing gingivitis or periodontitis, uni/bilateral lympho-epithelial cyst of the major salivary glands, cancrum oris, etc.), and also neoplasms (Kaposi’s sarcoma and Non-Hodgkin’s lymphoma) (Figure 2e) [14,15,16,17]. Although the overall incidence of such manifestations has strongly decreased with the introduction of HAART therapy, a periodic examination of the mouth of infected patients is strongly suggested for a careful control of the disease. 

**Figure 3:**
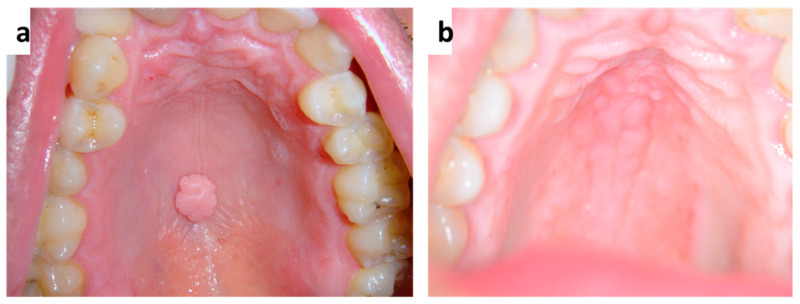
(**a**,**b**) Single (**a**) and diffuse (**b**) lesions of the palate related to Human Papillomavirus infection.

Human papillomavirus infection may cause several benign clinically papillary lesions in the oral cavity, such as squamous papilloma, (Figure 3a,b) condyloma acuminatum, verruca vulgaris, and multifocal epithelial hyperplasia (Heck’s disease), and also it could be associated to the occurrence of potentially malignant lesions and oro-pharyngeal squamous cell carcinoma [18,19,20,21,22,23,24]. Over two hundred human papillomaviruses may be spread vertically (maternal-fetal transmission), horizontally (between individuals by sexual, skin-to-skin, skin-to-mucosa, or mucosa-to-mucosa transmission) and by autoinoculation [25]. Virus penetration may surely be facilitated by mucosal breakings or micro-abrasions with the following infection of the basilar stem cells, which subsequently differentiate in keratinocyte with contextual genoma amplification, evasion of the immune system and virus diffusion by desquamation from the upper layers [25,26,27]. The clinical and differential diagnosis between squamous papilloma, condyloma acuminatum, verruca vulgaris is quite simple, but it mandatorily include giant cell fibroma, verruciform xanthoma, papillary squamous cell carcinoma, sialoadenoma papilliferum [28,29,30,31,32]. The diagnosis of multifocal epithelial hyperplasia is usually guided by clinical detection especially in children but, Cowden’s syndrome, neurofibromatosis type-1 and multiple endocrine neoplasia type 2B should always be considered in the differential diagnosis, along with the other benign clinically papillary lesions of the oral mucosa. 

**Figure 4:**
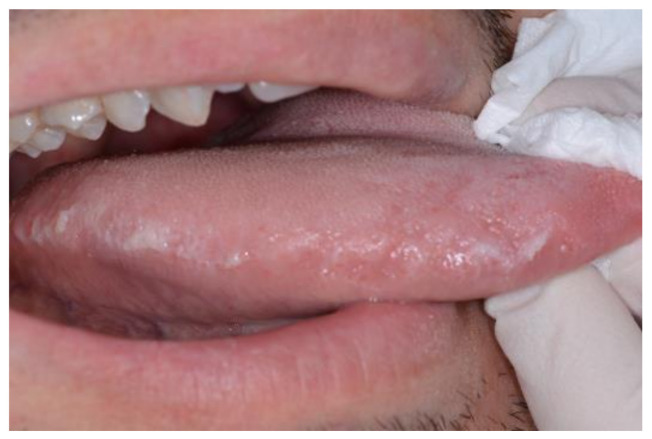
Hairy leukoplakia of the tongue margin associated to HBV infection.

Epstein–Barr virus (EBV) infection usually occurs at an early age and, as for all human herpesviruses of which EBV is referred to as HHV-4, is generally characterized by a viral latency in the B-cells of most adults after initial infection [32,33,34]. EBV causes infectious mononucleosis, hairy leukoplakia (Figure 4) also in healthy individuals, and, by a still not well defined tumorigenesis pathway of human oral epithelial or lymphoid tissue, several epithelial and non-epithelial neoplasms or tumor-like lesions in the head and neck, mostly in immunocompromised patients (such as Burkitt’s lymphoma, Hodgkin’s and non-Hodgkin’s lymphoma in immunodeficiency, post-transplant lymphoproliferative disorder, lymphoepithelioma-like carcinoma of the parotid gland, carcinoma of the nasopharynx) [35,36,37,38,39,40]. With the exception of infectious mononucleosis and hairy leukoplakia, which are quite frequent and relatively easily detectable and diagnosable by clinical examination, all the remaining EBV-related lesions/neoplasms represent a true dilemma; they are frequently detected at the advanced stage when the occur in the oro-maxillo-facial region since they are quite rare and clinical and radiological signs are not-specific [35,39].

## 3. Autoimmune and Disimmune Diseases

**Figure 5:**
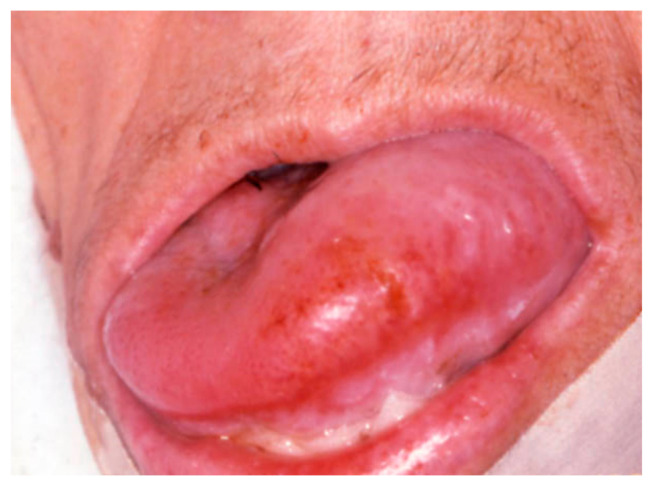
Macroglossia related to a nodular enlargement as first sign of Primary Amyloidosis.

Amyloidosis is a rare disease characterized by the deposition of insoluble aggregates of misfolded fibrillary proteins (amyloidogenic proteins) within the extra-cellular matrix of various tissues and organs, and by different forms (primary, multiple myeloma-associated and secondary) [41,42,43]. It occur in the head and neck approximately in the 40% of patients with systemic disease [44,45], mainly involving the larynx, orbit, sinus, salivary glands, while in the oral cavity the tongue (Figure 5) (nodular or diffuse enlargement, possible ulceration or hemorrhages) is the most frequently involved site, followed by the palate, and the gingiva [46,47,48,49]. Oro-facial lesions are frequently the first clinical manifestation and usually represent an unfavorable prognostic significance [43,50]. Diagnosis should be mandatorily confirmed by tissue biopsy and treatment is related to the stage of disease. 

Sjögren’s syndrome is an autoimmune disease with unknown etiology, mostly affecting women (female:male ratio 9:1) involving the exocrine glands (salivary and lacrimal glands) classified as primary or secondary (mostly associated to rheumatoid arthritis and other autoimmune diseases such as lupus erythematosus, sclerodermia, vasculitis, etc [51,52]. Common findings are keratoconjunctivitis sicca and xerostomia with thick, ropey and mucinous saliva or totally absent, atrophy of papillae and fissuring of the tongue, angular cheilitis mostly related to Candidiasis which is frequent in such patients [53]. In addition, all common symptoms related to the qualitative and quantitative reduction of saliva should be variably observable (dysphagia, taste alterations, difficulty speaking and chewing, etc.). Diagnosis is based on clinical, serological (serum circulating autoantibodies of SS are anti-SS-A/Ro and anti-SS-B/La) and histological (evaluation of the lymphocytic infiltrate surrounding the salivary ducts on samples of labial minor salivary glands) findings [54,55]. Nevertheless, most cases remain undiagnosed or diagnosed late. The potential evolution in MALT lymphoma observable in almost the 30% of patients affected by Sjögren syndrome along with the worsening of quality of life related to clinically advanced diseases, makes the early recognition of the disease extremely important to prevent complications [51,53,56].

**Figure 6:**
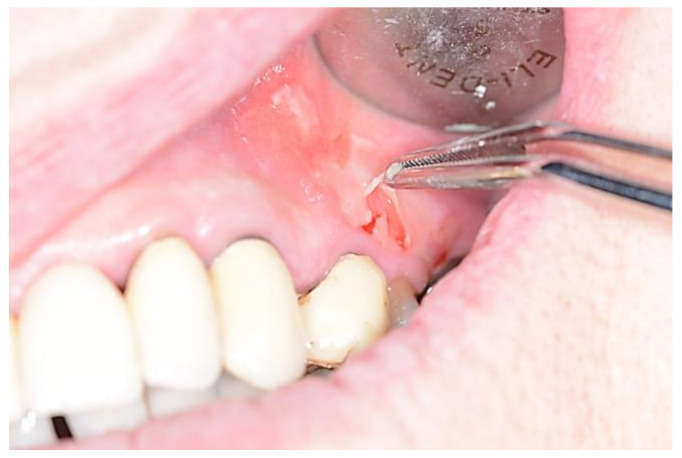
Nikolsky sign positivity in patient with pemphigus vulgaris.

Autoimmune blistering diseases are generally classified in Pemphigus and Pemphigoid group. Antigens and isotypes for each disease have been listed in Table 1. Pemphigus is a group of chronic autoimmune diseases caused by autoantibodies targeting specific desmosomal proteins on the surface of epithelial cells (in pemphigus vulgaris are typically linked to desmoglein-3 and rarely, in 50% of cases, also to desmoglein-1, the latter an exclusive target antigen in pemphigus foliaceus) with subsequent acantholysis and often with intraepithelial blistering [57,58]. Manifestations in the oral cavity are usually severe and frequently represent the first clinical sign of disease, usually appearing as painful and flaccid vesicles mostly of the gingiva, tongue, and palate with rapid evolution into erosion and ulceration, with the classic positivity to the Nikolsky sign (Figure 6) [59,60,61,62], Histological examination with adjunctive direct immunofluorescence of the tissues is mandatory for the differential diagnosis, along with blood test including BP180 e BP230. A clinical variant defined as Paraneoplastic pemphigus is often associated to a frequently not yet diagnosed neoplasm (mostly NHL, leukemia, multiple myeloma, etc.), characterized by intra- and sub-epithelial bullae but with a still unknown correlation between the underlying malignancy and autoimmunity [63]. Probably, an immune dysregulation with autoantibodies production (to desmoglein-3 but also to desmoplakins I and II, periplakin, envoplakin, BP230 [BPAg1], 170-kd membrane protein) occurs secondary to the neoplasm rather than as a primary cause). 

**Figure 7:**
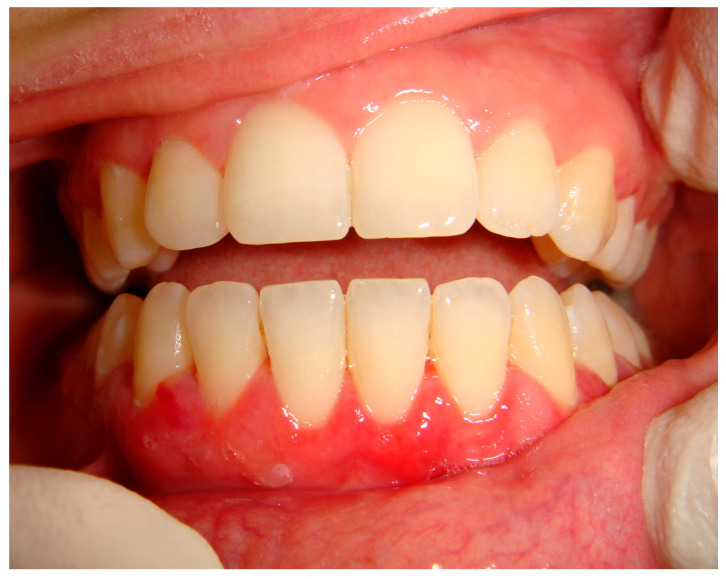
Diffuse desquamative gingivitis in the mandible unresponsive to the conventional medical and instrumental therapies, subsequently diagnosed as mucous membrane pemphigoid.

Pemphigoid group mainly includes bullous pemphigoid, dermatitis herpetiformis, mucous membrane pemphigoid, herpes gestationis, linear IgA bullous dermatosis and epidermolysis bullosa acquisita, but the two most involving the oral cavity are bullous pemphigoid and mucous membrane pemphigoid [57,58]. While bullous pemphigoid is the most common and the oral involvement occurs, usually synchronously to skin lesions, in the 10–20% of patients with gingival erosion, erythema or serous/hemorrhagic blisters, the clinical onset of mucous membrane pemphigoid is extremely variable [64,65]. In fact, patients, who are mostly female, may manifest oral lesions, especially desquamative gingivitis, (Figure 7) as initial and also single signs or associated to skin or other mucosal sites (mostly conjunctive with irritation, photophobia, ulceration, scarring-simblefaron). Bioptic tissues show a sub-epithelial bulla with scares inflammatory cells on H&E and linear deposits antibody of IgG and C3 along the epithelial-connective junction as resulting from autoantibodies production, mostly against Antigen BPAG2 (180 kD). Early diagnosis plays a pivot rule in such cases to prevent complications and to a rapid onset of the pertinent therapy. 

Behcet’s syndrome is a chronic inflammatory disorder characterized by the classic triad of oral, genital and ocular ulcers; additionally, a cutaneous as well vascular, central nervous system, gastrointestinal involvement may be variably observed. Oral lesions are frequently the first manifestation of the disease (as recurrent afta major, ischemic necrosis of the gingiva, etc.) [66,67]. Since no pertinent serological and/or bio-chemical activity markers exist for diagnosis and monitoring the disease, both are essentially based on the exanimation of lesions, symptoms and detailed medical history [68,69,70,71]. In fact, several systems for measurement (both scores and indexes) of patient-reported outcomes exist to better manage Behcet’s syndrome, some validate as disease-specific (Total Activity Index, Behcet’s Disease Current Activity Form, Behcet’s Syndrome Activity Scale) and other as organ-specific (Oral Ulcer Severity Score, Genital Ulcer Severity Score, Composite Index for oral ulcers and Muco-cutaneous Index) [72,73]. Overall, occurrence of mucocutaneous lesions (which is classically in women and decrease with advancing age) with musculoskeletal involvement represents the milder manifestation of the disease; oral ulcer activity, which is usually persistent especially in young male, and frequently precedes other organ involvement [66,67,68,72,73,74].

**Figure 8:**
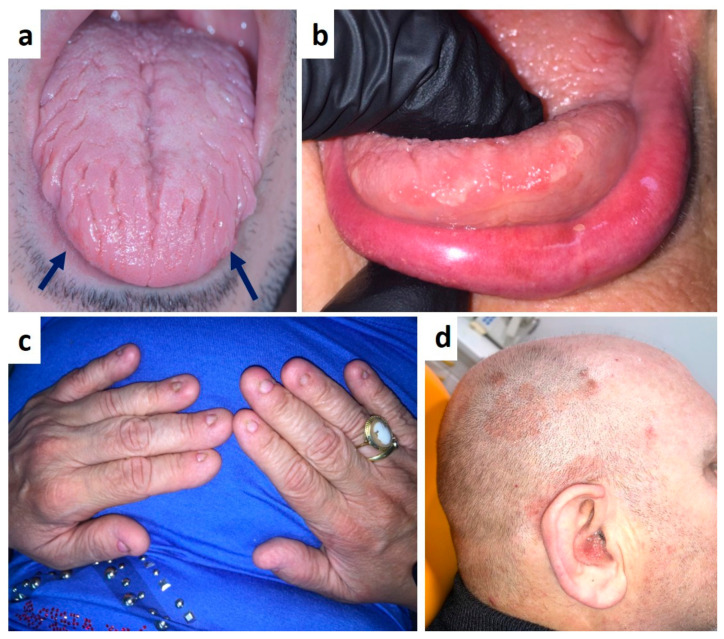
(**a**) Fissured tongue and early eritro-leukoplakia like lesions (arrows) in patient with no cutaneous psoriasis. Round white-yellowish lesions of the tongue apex and white lesions of the lip in patient with familial history of psoriasis (**b**). Anonychia in patient with a long-lasting duration history of psoriasis (**c**) and the classic cutaneous lesions of the head skin (**d**).

Psoriasis is a chronic inflammatory muco-cutaneous disease predominantly affecting the skin with various clinical presentations [75]. The intraoral presentation, either coexisting with cutaneous lesions or presenting as isolated oral lesions, is rarely reported in the literature; oral mucosa and other mucous membrane involvement may occur especially in association with generalized pustular or erythrodermic variants of psoriasis [76,77,78,79]. Common findings in the oral cavity are leukoplakia-like lesions mostly of the cheek, palate, desquamative gingivitis, migrant glossitis or geographic/fissured tongue [76,78]. Nevertheless, the existence itself of psoriasis manifestations in the oral cavity is still controversial. In fact, although it is generally accepted that patients with psoriasis manifest oral lesions synchronously with skin disease, both showing similar histopathological features and a parallel clinical course, a positive family history and positive HLA typing for either B13, B17, B37, Cw4, or Cw6 genes are generally used as diagnostic criteria [76,78,79]. Oral lesions showing diagnostic histological features, in absence of contextual skin involvement, are usually attributable to a remission phase of a cutaneous psoriasis. (Figure 8a–d) [80,81,82], The lack of the aforementioned diagnostic criteria, lead several authors to consider suspicious lesions as psoriasiform mucositis rather than true oral psoriasis [78,83,84]. Therefore, the true incidence of oral involvement in psoriasis is still unknown and this is surely related its rarity, the peculiar transient nature of oral lesions, absence of clinical and histopathological general consensus to the diagnosis of oral psoriasis. However, it cannot be excluded that poor attention is usually paid to the oral cavity of patients with a frank cutaneous psoriasis or only a familiar history of psoriasis and above all a mucosal biopsy is carried out in very few cases [85].

**Figure 9:**
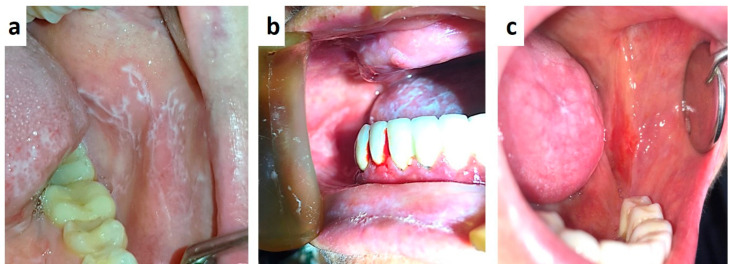
(**a**–**c**) Reticular white lesions of the cheek mucosa as early manifestation of Lichen Planus (**a**). Diffuse white lesions of the tongue, lip and adherent gingiva, eritro-leukoplakia of the cheek mucosa and diffuse autoimmune gingivitis in the mandible in patient with severe disease still untreated (**b**). Eritroplakia of the cheek detected during follow-up in a patient with Lichen Planus of long-lasting duration (**c**).

Lichen planus (LP) is a common muco-cutaneous disease that can be remain confined exclusively to the oral mucosa or present in a more widespread format [86,87,88]. It is an autoimmune/inflammatory condition affecting the skin, oral mucosa and tongue; within the oral cavity it may manifest in several different forms or subtypes generally described as reticular or ‘lace like’ (Wickham’s striae), bullous and erosive [86,89]. LP lesions, especially in the reticular form, are often asymptomatic and are usually found during dental check-up. Symptoms are instead referred by patient showing erosive lesion related to LP. Such patients, when the diagnosis is confirmed by histological findings, should be monitored to prevent malignant transformation too [89,90,91]. In fact, changes in clinical presentation of the intra-oral lesions during follow-up, which can last several years or even decades, required careful attention and, often, additional biopsies to exclude dysplasia or malignancy. (Figure 9 a–c). Symptom control and/or regression of oral lesions may be achieved by using topical and/or systemic corticosteroid. Although the clinical diagnosis is relatively easy, the panel of differential diagnosis is wide as including lichenoid drug reactions, oral psoriasis, graft-versus host disease, lupus erythematous discoid, frictional cheratosis, candidiasis, eritroplakia, oral carcinoma at the early stage; association with other systemic diseases, mostly Hashimoto thyroiditis, should be always investigated in LP patients.

**Figure 10:**
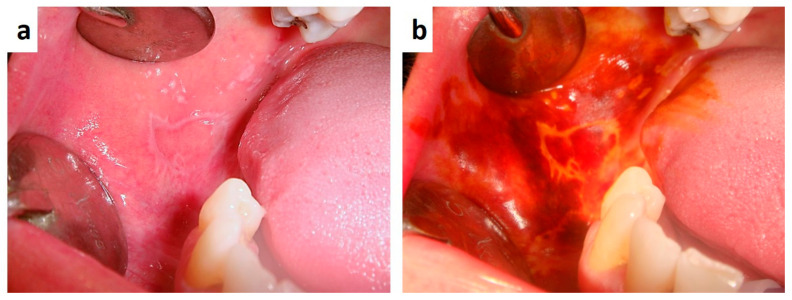
(**a**,**b**) The detection of an erythematous lesion with radiating white striae of the cheek (**a**), better highlighted by vital die with Lugol’s solution (**b**), is frequently the first clinical manifestation of a still undiagnosed discoid lupus erythematosus.

Lupus erythematosus usually refers to a group of autoimmune diseases mostly involving the skin but with systemic manifestation too [92]. Systemic lupus erythematosus (SLE) and discoid lupus erythematosus (DLE) present with oral findings in 8–45% and 4–25% of patients, respectively [93,94]. SLE is the most common vascular collagen disorder and frequently manifests associated oral lesions with an high clinical variability as ulcerations, erythema or hyperkeratosis, and often cheilitis may also be observable [92]. DLE-related oral findings are usually ulcerated, atrophic, and erythematous lesion of the oral mucosa; the classic clinical intra-oral appearance is a central erythematous area with radiating, fine and white striae mainly resembling erosive lichen planus, (Figure 10) while round, reddish with white striae, target-like lesions may involve the head and neck cutis [92,93,94]. Diagnosis is relatively easy in patient with an already diagnosed systemic disease; usually, oral lesions typically resolve with systemic therapy immunosuppressive, topical corticosteroids and systemic antimalaric drugs. conversely, in undiagnosed patients, the differential diagnosis of oral lesions may be challenging as based on clinical, hematological and histological findings. Also, considering the potentially malignant transformation of LED, the oral biopsy should be carefully performed possibly also in multiple sites and histological examination aimed to evaluate the possible presence of epithelial dysplasia or early carcinoma [95].

**Figure 11:**
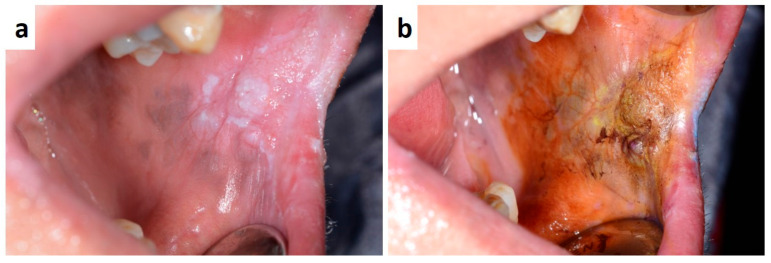
Non-homogenous hyperkeratotic patches with partial ulceration of the cheek and labial commissure following allogenic hematopoietic stem cell transplantation (**a**); a careful clinical examination with the use of vital dies (toluidine blu and Lugol’s solution), (**b**) highlighted area of potential malignant transformation, subsequently diagnosed as epithelia dysplasia and early squamous cell carcinoma.

Graft Versus Host Disease (GVHD) is a severe form of erosive stomatitis occurring in patients affected by blood malignancies, bone marrow and lymphoid system as well systemic immunodeficiencies, and treated by allogenic hematopoietic stem cell transplantation, as consequence of an immunological attack by donor T cells transferred with the graft. The degree of morbidity of GVHD patients varies from mild to severe and clinical manifestations may variable involve the skin, mouth, eyes, gut, liver, joints, lungs. Oral lesions are very frequent as occurring in approximately 45% to 83% of patients [96], and may involve the mucosa, the salivary gland and also the perioral tissues by sclerotic involvement. Common intraoral findings in GVHD patients are essentially lichenoid changes of the oral mucosa, erythematous or ulcerated lesions, hyperkeratotic or atrophic mucosal patches, mostly resembling lichen planus and proliferative verrucous leukoplakia (Figure 11a,b) [97,98], Limited mouth opening or salivary gland dysfunction may be related to direct organ involvement and/or to fibrotic or sclerotic involvement of tissues, thus resembling several autoimmune diseases with oral and maxillofacial manifestations, including SS and scleroderma [96,97,98]. GVHD patients should be periodically and carefully screened for possible occurrence complications related to the drugs and immunosuppression such oral candidosis and oral squamous cell carcinoma, and suspicious lesions immediately biopsied during the entire follow-up [99,100].

## 4. Granulomatous Disease

The term oro-facial granulomatosis encompasses several conditions exhibiting similar clinical (mostly persistent enlargement of the soft tissues of the oral and maxillofacial region) and microscopic features (presence of non-caseating granulomas on histological examination), often associated with systemic conditions such as sarcoidosis and Crohn’s disease [101,102,103,104,105,106].

Sarcoidosis is a multi-system granulomatous disease of unknown origin, usually affecting the chests of young and middle-aged adults by bilateral hilar lympho-nodes involvement [101,102,107,108]. Skin, muscle, nerves, liver, heart, kidney and joints may be variably involved by formation of non-caseating giant-cell granulomas. In the head and neck, Sarcoidosis may involve salivary glands and lateral neck lympho-nodes manifesting as asymptomatic and low-growing swellings, while single or multiple submucosal nodules, although un-specific and rare, may be detectable in the lip and cheek [107]. A peculiar clinical form of Sarcoidosis is the Lofgren’s syndrome, characterized by salivary and lacrimal gland swelling, uveitis and peripheral palsy of the facial nerve [101,102,109]. The diagnosis is based on chest radiography, hematological investigations with Angiotensin-Converting Enzyme dosage, incisional or core-needle biopsy of lesions confirming the presence of non-caseating granulomas formation. 

**Figure 12:**
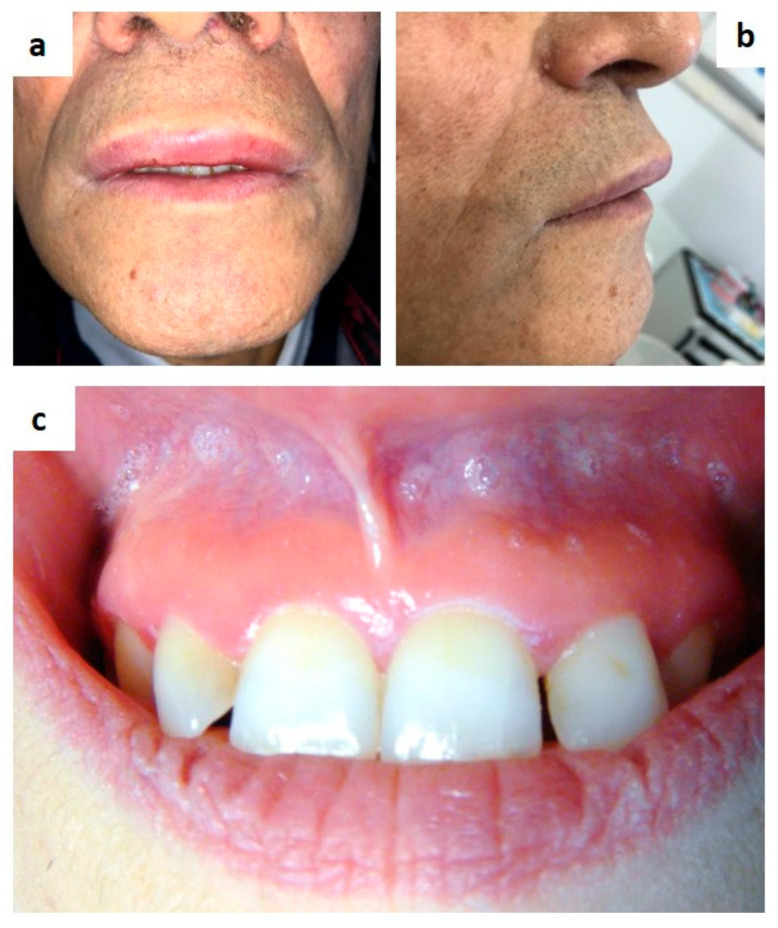
(**a**–**c**) Classic upper lip swelling in patient with oro-facial granulomatosis (**a**); the gingival onset as diffuse gingivitis (mostly of the maxillary adherent gingiva and unrelated to plaque or calculus) is reported to be very rare in unaware patients.

Melkersson-Rosenthal syndrome is an oro-facial granulomatous disease classically characterized by a triad of symptoms such as oro-facial swelling (mostly of the lip, Figure 12a) recurrent peripheral facial nerve palsy and fissured tongue. Such clinical signs may occur synchronously or metachronously also after many years [103,110,111]. When just the swelling of the inferior lip is detectable, the disease is known as granulomatous cheilitis or Miescher’s disease. Also, swelling of the tongue, cheek and palate may be present, and diffuse erythema of the gingiva unresponsive to medical/instrumental treatments. (Figure 12b,c) The diagnosis is generally achieved by clinical sign detection and histological examination of the involved tissue, the latter useful for the differential diagnosis with angioedema, Crohn’s disease, amyloidosis [106].

Wegener’s granulomatosis (WG) (or granulomatosis with polyangiitis) is a very rare, life-threatening systemic disease with a not-completely yet defined etiology [112]. The pathogenesis is immune-mediate with vasculitis of small/medium caliber vessels and formation of auto-antibodies anti-neutrophil cytoplasma/c-ANCA) which is a hematological finding specific for the disease [112,113,114]. WG lesions are characterized by a triad of phase such as necrosis, granulomatous inflammation and vasculitis, with damage of the involved sites/organs. Upper respiratory tract, lungs and kidneys are the most involved sites, although can affect multiple systems in the whole organism. Head and neck signs of the disease are chronic sinusitis with or without pus or bloody drainage, salivary gland swelling, nasal and oral mucosa ulcers, tongue necrosis, facial palsy, but the most reported clinical presentation is as gingival hyperplasia, most precisely a strawberry-like gingivitis/lesion of the maxilla [112,113,114,115]. The differential diagnosis includes angioedema, Crohn’s disease, other granulomatosis, amyloidosis, NHL (mostly NK type) and oral squamous cell carcinoma too [112].

**Figure 13:**
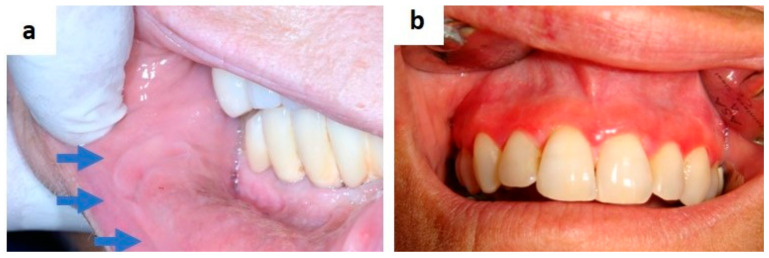
(**a**,**b**) Mucosal tags of cheek in patient with Chron’s disease (**a**); persistent and diffuse erythematous gingivitis at the maxilla as first manifestation of the disease in absence of intestinal symptoms (**b**).

Crohn’s disease (CD) is a chronic, systemic, immune-mediated inflammatory bowel disease that frequently exhibit extra-intestinal manifestations, including oro-facial signs in both adults and children. Clinical oral signs of CD are usually distinguished in specific (diffuse lip and buccal swelling, tags, cobblestones) and not-specific (aphthous ulcers, pyostomatitis vegetans, and gingivitis) (Figure 13a,b) [116,117], In patient showing one or more of the lesions, the biopsy is mandatory to achieve final diagnosis and for the differential diagnosis mostly with foreign-body reactions, sarcoidosis, mycobacterial infection and fungal sepsis. Overall, oral lesions may be variably identified in up to 60% of patients, while in 5–10% of cases they represent the first manifestation of a still unknown CD [101,102,118]. The oral manifestations of CD are also uncommon in children and can precede or coincide with intestinal inflammatory lesions, leading frequently to a delayed diagnosis [119,120,121].

## 5. Drug-Induced Oral Lesions

**Figure 14:**
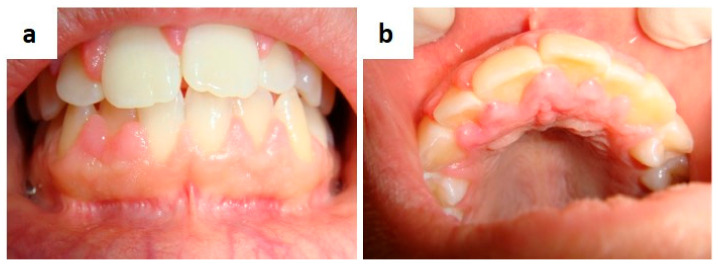
(**a**,**b**) Anticonvulsants-induced gingival overgrowth in young involving the interdental papillae and the adherent gingiva of both jaws.

Drug-induced gingival overgrowth (DIGO) is a side-effect related to the systemic therapy by drugs with no direct action on gingival tissue, mainly anticonvulsants, immunosuppressants, and calcium channel blockers; it can manifest in patients in close relationship to several general factors (age, genetic predisposition, duration of therapy) or local predisposing conditions (preexisting plaque/calculus and gingival inflammation) [122,123]. As for pathophysiology, the inhibition of cation influx, particularly sodium and calcium ions, is described as the drug-related mechanism of action [124]. DIGO mainly involves the anterior gingival tissue but it could be diffuse to the entire adherent gingiva also of both jaws (Figure 14a,b). Gingival edema and bleeding are always present because the difficulties to perform a proper dental hygiene, especially for the formation of gingival pseudo-pockets with subsequent plaque and calculus accumulation; edentulous area are typically not involved by DIGO [124,125]. Although the diagnosis appears relatively simple, the differential diagnosis scenario is extremely wide as includes hematological disorders, head and neck syndromes, genetic diseases, neoplastic and inflammatory lesions of the gingiva [125,126]. Suspension/modification of drug therapy along with oral hygiene maintenance, possibly improved by laser gingival decontamination, are mandatory in such patients promoting an impressive reduction of the overgrowth and often resolution; conventional surgical reduction or laser treatment of the gingival mass remain decisive for persistent enlargements [126,127].

**Figure 15:**
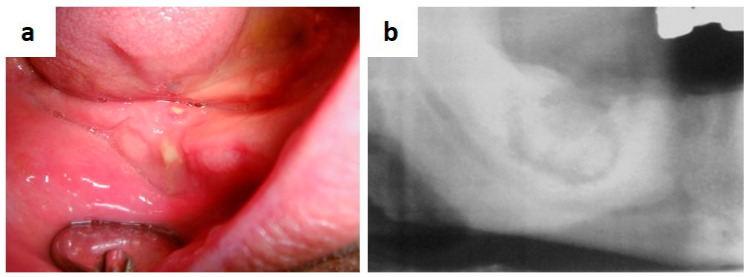
(**a**,**b**) Post-extraction site with delayed healing, swelling, pus discharge and bone exposure (**a**) in patient taking Zoledronic acid (e.v.) as in systemic therapy for multiple myeloma; lesion appears as a radiolucency with ill-defined borders encompassing a bone sequestrum (**b**).

Medication-related osteonecrosis of the jaw (MRONJ) is a side-effect of systemic drug therapy such as bisphosphonates, denosumab, bevacizumab and sunitinb [128,129]. The American Association of Oral and Maxillofacial Surgeons defined MRONJ as “*presence of exposed necrotic bone or bone that can be probed through an intra- or extraoral fistula in the oral cavity, or bone that can be probed through an intraoral or extraoral fistula in a patient currently taking or previously treated with antiresorptive or antiangiogenic drugs who never underwent radiotherapy for head and neck neoplasms, persisting for more than 8 weeks after clinical identification*” [130]. Early signs of disease are maxillary or mandibular sites (mainly post-extractive, but also perimplant site or associated to endodontic sequelae) with delayed healing and persistent mucosal incompetence, following by superinfection and bone sequestrum. (Figure 15a,b) [130,131,132,133], Lesions may be single or multiple, synchronous or metachronous necrotic areas of the jaw, with pus drainage and with or without bone exposure. Overall, any dental procedure may represent a risk factor for MRONJ in patients taking the aforementioned drugs [134]. Biopsy is mandatory in all instances to achieve a diagnosis and also for the differential diagnosis, the latter mostly including secondary malignant localizations (including metastasis), primary benign or malignant odontogenic or bone neoplasms.

## 6. Hematologic Disorders

Hematologic disorders/malignancies and blood cell dyscrasias are a wide group of diseases with systemic dissemination which may variably involve the oral cavity and, overall, the hard and soft tissues of head and neck, frequently representing the first clinical manifestation. In addition, several complications of medical immunosuppressive treatments for such diseases may involve the oral mucosa, such as viral/fungal/bacterial infection, mucositis, Candidiasis.

Acute leukemia most commonly manifests oral lesions than chronic leukemia, especially with edematous and erythematous gingival hypertrophy/hyperplasia. Petechiae, ecchymoses of the oral mucosa and spontaneous gingival bleeding are common manifestation of thrombocytopenia [135].

As for lymphomas, while the oral involvement by Hodgkin lymphoma is relatively un-frequent, instead, non-Hodgkin lymphoma (including the MALT lymphomas), frequently manifests, and often at the onset, in the oral cavity as painless, soft and usually slow growing masses/swellings of the Waldayer ring, palate, gingiva, buccal mucosa, tongue, also with superficial ulceration mostly related to trauma; neck lympho-nodes are frequently involved too [136,137,138].

Multiple myeloma manifests frequently oro-facial signs as osteolytic lesions principally involving the mandible with swelling, pain and additionally paraesthesia and tooth loss related to bone destruction.

Langerhans cell histiocytosis is a rare hematologic disorder usually affecting children and most commonly (55% to 80% of cases of disseminated disease) involving the head and neck region, although rarely as primary site. Signs and symptoms differ according to the unifocal or multifocal clinical occurrence of LCH [135,136,137,138]. The skull (mostly of the temporal bone), lymph nodes, skin and orbit, are frequently involved while oral lesions (mostly of the jaws) are essentially localized or multiple gingival swellings with edema/inflammation. periodontal involvement, teeth mobility/dislocation, destroying bone lesions often with the classical appearance of “floating teeth on radiograms [139,140,141,142,143]. Less frequent is the onset of a single bone LCH lesion (especially of the maxilla) with punch-out radiolucent appearance and cortical bone erosion or diffuse periodontal involvement and mucosal infiltration [144]. Early diagnosis is challenging as LCH lesion in head and neck mimics most common pathologies (especially periodontal diseases), often resulting in a diagnostic delayed [145].

## 7. Endocrine Diseases (Miscellanea)

The close relationship between diabetes and the oral cavity is well recognized; decreasing salivary gland function is the main diabetes-related alteration, also leading to variable degree of xerostomia thus increasing the possible occurrence of fungal/bacterial infections of the mouth as well taste alterations. Gingivo-parodontal manifestations are high occurrence of dental caries, gingivitis, periodontitis, gingival bleeding often massive, burning mouth symptoms, oral ulcers and lichen planus too [146,147].

Hypercorticoadrenalism (primary or Addison’s disease and secondary) is caused by progressive destruction of the adrenal cortex with consequent insufficient secretion of adrenal androgens, mineralocorticoids and glucocorticoids. Along to the systemic signs and symptoms variably related to the reduced secretion functions (weakness, fatigue, loss of appetite and weight) pigmentation of the skin (also of the head and neck) and of the oral mucosa are common findings [148]. The latter, as easily detectable, frequently arise the suspicion of disease in still undiagnosed patients. However, the differential diagnosis includes other pigmentations of the oral mucosa also associated to the Peutz-Jeghers and McCune-Albright syndromes.

Systemic manifestations of hyperparathyroidism may involve bones, kidneys, soft tissues, the gastrointestinal tract, the central nervous system, the odontogenic tissue and the jawbones [149,150,151]. Giant cell lesions (GCLs) of the oral and maxillofacial region associated with hyperparathyroidism (HPT) are rare clinical entities [151,152]. They are also called brown tumours because of their clinical appearance and may mimic other peripheral or central giant cell lesions clinically, radiologically and histologically too. Complete or partial loss of lamina dura and a ground glass appearance of the jaw bones, occasionally found on radiograms in most cases, are the peculiar findings. GCLs formation usually represents the terminal stage of bone disease related to HPT, as resulting from an imbalance of osteoclastic and osteoblastic activity leading to resorption with fibrous replacement of the bone [153]. Although mandibular involvement has been reported with an incidence of 4.5% of subjects it is quite rare the identification of a GCL as the first clinical manifestation of primary HPT before the onset of general manifestations [151,152,153]. An accurate screening is mandatory in HPT patients especially when of long-lasting duration. Radiolucent lesions of the jaws showing giant cells on histopathologic examination after incisional or excisional biopsy should always raise suspicion of HPT also in asymptomatic patients by an accurate clinical and diagnostic work-up. It is generally accepted that histological findings alone are insufficient for diagnosis which is definitively confirmed by establishing elevated serum calcium and parathyroid hormone levels.

Multiple endocrinous neoplasia (MEN) syndrome is characterized by the occurrence in the same patient of tumours (glandular hyperplasia and/or malignancy) of two or more endocrine glands, generally classified into four major forms (from MEN1 to MEN4) [154,155,156]. All the four forms may be inherited as autosomal-dominant syndromes or sporadically occur as detected in patients with a missing family history, probably because of the death of familiars before symptoms developed [156,157,158]. MEN1, also known as Wermer’s syndrome, is characterized by the concomitant occurrence of neoplasms of the parathyroid glands, pancreatic islet cells, and the anterior pituitary; additional tumours (adrenal cortical tumours, carcinoid, collagenomas, lipomatous tumours and facial angiofibromas) have been also reported [156,157]. MEN2, previously classified as MEN2A and also called Sipple’s syndrome, is characterized by occurrence of medullary thyroid carcinoma associated to phaeochromocytoma and parathyroid tumours. MEN3 (previously referred to as MEN2B) is characterized by occurrence of medullary thyroid carcinoma and phaeochromocytoma in association with marfanoid habitus, pectus excavatus, medullated corneal fibers, and intestinal dysfunction with megacolon, cutaneous (including the eyelid) and mucosal neuromas also of the larynx and oral mucosa [154,155,156,157,158]. Oral lesions are multiple, nodular and sessile lesions mainly of the tongue, lip and the cheek, usually covered by a normo-coloured mucosa [156]. Their detection within the oral cavity is relatively easy but the differential diagnosis panel is wide, as including multiple neuorofibomas in neurofibromatosis type-1, multiple papillomatosis, traumatic or reactive lesions and amyloidosis too.

## 8. Genetic Diseases and Head and Neck Syndrome (Miscellanea)

The clinical, diagnostic and genetic scenario of such diseases is extremely wide, also including the variable association of sign and symptoms for the differential diagnosis. Among all, *Peutz-Jeghers syndrome, Gorlin syndrome, and Gardner syndrome* are surely the most frequently diagnosed by early oral and head and neck signs/lesions.

**Figure 16:**
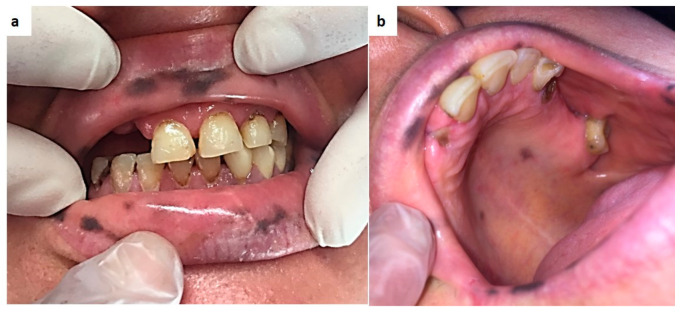
(**a**,**b**) Diffuse hyperpigmentations of the lips and oral mucosa in Peutz-Jeghers syndrome patient.

In fact, Peutz-Jeghers syndrome is a rare autosomal dominant disorder, often undiagnosed for years or accidentally diagnosed by occurrence of acute symptoms such as bleeding, bowel obstruction and intussusception, just because it is characterized by gastrointestinal hamartomatous polyps and hyperpigmentation on the lips, check and often of the entire oral mucosa (Figure 16a,b) [159,160,161].

Gorlin syndrome, or basal cell nevus syndrome, is a hereditary disease related to the mutations (more than 100 gene mutations have been reported) of *patched 1*, *patched 2* and *PTCH2*, and *SUFU* genes, characterized by systemic and diverse developmental abnormalities and neoplastic lesions [162,163,164]. Patients may variably display multiple basal cell carcinomas (also of the head and neck skin), medulloblastomas, frontal ridges, coarse facial features, facial milia, skeletal abnormalities (bisecting ribs and wedge-shaped vertebrae), intellectual disability, heart and ovarian fibromas, palmar and/or plantar pits hyperkeratosis [163,164]. As for head and neck signs/symptoms, patients may be affected by ectopic calcification (especially of the cerebral falx), eye abnormality, lip or palate cleft, single or multiple odontogenic keratocysts of the jaw which are frequently the early sign of the disease and often occasionally detected in paediatric patients [165,166,167].

**Figure 17:**
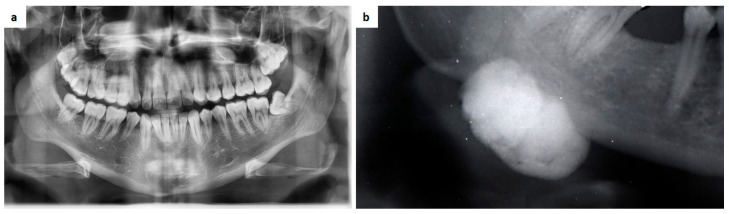
(**a**,**b**) Supernumerary teeth in the maxilla and multiple focal increases of bone density may represent the early clues of an undiagnosed Gardner syndrome especially in young (**a**); large mandibular osteoma in an already diagnosed patient (**b**).

Gardner syndrome is a variant of familial adenomatous polyposis syndrome characterized by numerous colonic polyps with prominent extra-colonic manifestations, such as skull and jaw osteomas and various soft-tissue tumours (overall, fibromas, neuro-fibromas, sebaceous cysts, leiomyomas, lipomas, desmoid tumour; rarely, papillary carcinoma, adrenal adenoma, adenocarcinoma, hepatocellular carcinoma, osteosarcoma, chondrosarcoma, osteochondroma, thyroid and liver tumours and hypertrophy of retinal pigment epithelium have also been reported [168,169,170]. More than 50% of patients with familial adenomatous polyposis show osteomas in the oral and maxillofacial region (jaw and paranasal sinuses) which can considered typical signs. Less frequently is the association with odontomas, supernumerary teeth, focal increases in bone density within the jaws but such signs, often occasionally detected in not-still diagnosed patients or in young with an already known or not familial history, in the majority of cases represent the early clues of the disease (Figure 17a,b) [169,170,171,172].

## 9. Metastases to the Oro-Facial Tissues

Metastases to the oro-facial tissues are very rare as their incidence ranges between 1–8% of all oral malignant tumours, mostly occurring in the 5th–7th decades of age [173,174,175]. The most frequent primary tumour occurs in the lung, kidney, prostate and colon-rectum in males, while in the breast, lung, uterus and ovary in females. Oro-facial metastases to the tissues can involve the oral mucosa, jawbones or the salivary glands may be variably involved also as first sign of a still occult cancer or manifestation of disseminated disease [176,177,178]. Also, a predilection of metastases for specific sites in the oro-facial region is generally well recognized (especially the molar and premolar regions by virtue of their richer vascularization and higher bone marrow content); this is also probably influenced by peculiar clinical conditions of oral hard and soft tissue, such as parodontal tissues with inflammatory lesions of dentates, or gingival tissues in edentulous individuals bearing prosthesis, or post-extractive sites probably as a consequence of increased blood flow following organization of the blood cloth [174,178,179,180]. Clinically, oro-facial metastases classically manifest as rapidly growing lesions of the gingiva associated or not to bone involvement with the radiological appearance of aggressive neoplasms, or facial swelling when occurring in the major salivary glands [173,174,180]. Their recognition, especially in undiagnosed or occult primary cancers, represents a true dilemma for clinician as the differential diagnosis (clinical and radiological) is very challenging, and for the pathologist too in the proper definition of the primary tumour by sample analysis, especially when the diagnostic work-up is limited to the head and neck.

## 10. Conclusions

The clinical scenario of the oro-facial manifestations of systemic diseases is very wide, especially considering the possibility that they may manifest a parallel course to the systemic lesions, occurring synchronously or metachronously also preceding them and, in addition, as first clinical sign in unaware or still undiagnosed patients. Surely, an overall diagnostic delay is frequently associated to the therapeutic course in such patients as clinical recognition may result difficult as well the proper histological identification of sampled lesions. Hence, dentists and general practitioners should be clinically and academically educated to achieve a timely diagnosis by identification of the most common or the specific oro-facial manifestations pointing to a systemic or generalized disease.

## Figures and Tables

**Table 1:** Main autoantigens in pemphigus and pemphigoid.

**Pemphigus**	Target Antigens *	Ig **
Vulgaris	Dsg 3 in mucosal PV, Dsg 1 and 3 in muco-cutaneous PV	IgG
Foliaceus	Dsg 1	IgG
Paraneoplastic	Envoplakin, periplacin, Dsg 1/3, BP180, others	IgG
IgA	Dsg 1/3, Dsc 1–3	IgA
Herpetiform	Dsg 1	IgG
**Pemphigoid**		
Bullous	BP180-NC16A, BP230	IgG/IgE
Mucous membrane	BP180, BP230, laminin-332, α4β6 integrin, laminin-331, COL7	IgG
Gestationis	BP180-NC16A	IgG
Linear IgA disease	LAD-1	IgA
Epidermolysis bullosa acquisita	COL7	IgG/IgA
Anti-p200	Laminin γ1	IgG
Lichen planus pemphigoides	BP180-NC16A, BP230	IgG

*^,^** The main target antigens (also excluding the non-Dsg as rare) and the main isotypes have been listed. Legend: Dsg, desmoglein; Dsc, desmocollin; COL7, type VII collagen; LAD-1, linear IgA disease antigen-1.
